# Antibiotic use for Australian Aboriginal children in three remote Northern Territory communities

**DOI:** 10.1371/journal.pone.0231798

**Published:** 2020-04-17

**Authors:** Timothy Howarth, Raelene Brunette, Tanya Davies, Ross M. Andrews, Bhavini K. Patel, Steven Tong, Federica Barzi, Therese M. Kearns

**Affiliations:** 1 Child Health, Menzies School of Health Research, Darwin, Northern Territory, Australia; 2 Child Health, Sunrise Health Service Aboriginal Corporation, Katherine, Northern Territory, Australia; 3 Public Health and Planning, Sunrise Health Service Aboriginal Corporation, Katherine, Northern Territory, Australia; 4 Tropical Health, Menzies School of Health Research, Brisbane, Queensland, Australia; 5 Medicines management—Research, Transformation and Change, Top End Health Service, Darwin, Northern Territory, Australia; 6 Doherty Institute for Infections and Immunity, University of Melbourne, Melbourne, Victoria, Australia; 7 Wellbeing and Preventable Chronic Diseases, Menzies School of Health Research, Darwin, Northern Territory, Australia; UNSW Sydney (formerly University of New South Wales), AUSTRALIA

## Abstract

**Objective:**

To describe antibiotic prescription rates for Australian Aboriginal children aged <2 years living in three remote Northern Territory communities.

**Design:**

A retrospective cohort study using electronic health records.

**Setting:**

Three primary health care centres located in the Katherine East region.

**Participants:**

Consent was obtained from 149 mothers to extract data from 196 child records. There were 124 children born between January 2010 and July 2014 who resided in one of the three chosen communities and had electronic health records for their first two years of life.

**Main outcome measures:**

Antibiotic prescription rates, factors associated with antibiotic prescription and factors associated with appropriate antibiotic prescription.

**Results:**

There were 5,675 Primary Health Care (PHC) encounters for 124 children (median 41, IQR 25.5, 64). Of the 5,675 PHC encounters, 1,542 (27%) recorded at least one infection (total 1,777) and 1,330 (23%) had at least one antibiotic prescription recorded (total 1,468). Children had a median five (IQR 2, 9) prescriptions in both their first and second year of life, with a prescription rate of 5.99/person year (95% CI 5.35, 6.63). Acute otitis media was the most common infection (683 records, 38%) and Amoxycillin was the most commonly prescribed antibiotic (797 prescriptions, 54%). Of the 1,468 recorded prescriptions, 398 (27%) had no infection recorded and 116 (8%) with an infection recorded were not aligned with local treatment guidelines.

**Conclusion:**

Prescription rates for Australian Aboriginal children in these communities are significantly higher than that reported nationally for non-Aboriginal Australians. Prescriptions predominantly aligned with treatment guidelines in this setting where there is a high burden of infectious disease.

## Introduction

Antibiotics have saved millions of lives since modern refinements of ancient Chinese, Roman and Greek preparations were undertaken in the early 1900s. [1] However, in this modern era the overuse of antibiotics has resulted in the emergence of antimicrobial resistance (AMR) that poses a significant threat to public health. [1, 2] By the year 2050, AMR is expected to directly result in 10 million deaths per year. [3] Of particular concern is Australia’s antibiotic use, which is ranked eighth for the highest use of antibiotics among the Organisation for Economic Co-operation and Development (OECD) nations. [4] In response, the Australian government introduced the First National Antimicrobial Resistance Strategy 2015–2019, with an aim to combat the overuse of antibiotics and subsequent resistance. [5] As part of this strategy yearly publications describing Antimicrobial Use and Resistance in Australia (AURA) have been produced. [2, 5]

In 2015, data from the Pharmaceutical Benefits Scheme (PBS) indicated 51% of Australian children aged 0–4 years had been prescribed antibiotics. [2] However, this data does not capture remote primary health care (rPHC) services, who provide a significant proportion of primary health care (PHC) to Aboriginal people living in remote locations in Australia. In the Northern Territory (NT), rPHC services are provided predominantly by Remote Area Nurses (RANs) and Aboriginal Health Practitioners (AHPs), who conduct physical assessments and supply and administer antibiotics in accordance with the Central Australian Remote Practitioners Association Standard Treatment Manual (CARPA STM) [6]. Antibiotics are supplied in bulk to the PHC services under the Remote Area Aboriginal Health Services program and are thus not captured under the National PBS datasets. [7] The only record of who received these antibiotics is held in the local PHC electronic health record.

Evidence suggests Australian Aboriginal children are prescribed antibiotics at a higher rate than non-Aboriginal children of the same age. [8, 9] This is likely due to increased prevalence of infectious diseases requiring antibiotics, a factor which is further heightened among Aboriginal children living remotely in the NT. [9, 10] PHC presentations for impetigo and otitis media (OM) are frequent with 87% of children aged <24 months and 91% aged 6–30 months attending a PHC service for which antibiotics were prescribed in 49% [11] and 48% [12] of cases respectively. Furthermore, it has been suggested that increased sequalae risk may result in a lowered prescribing threshold. [13] Despite the increased prevalence and risk from infections, it has been suggested that the appropriateness of antibiotic prescriptions is higher in remote communities compared to urban areas. [9]

There is a significant dearth of data on prescription rates and prescription appropriateness for antibiotic use in rPHC services. With AMR increasing, surveillance is needed in these communities who, with an already high infection prevalence, are at particular risk. We report on antibiotic prescriptions in the first two years of life for children born between 1 January 2010 and 31 July 2014 living in three remote NT Aboriginal communities.

## Methods

### Study design and setting

We conducted a retrospective cohort study to report on antibiotic prescriptions by remote health practitioners for Aboriginal and/or Torres Strait Islander children born between 1 January 2010 and 31 July 2014 in three remote NT Aboriginal communities located in Katherine East region, Australia. This study was embedded in a larger mixed-methods evaluation of anaemia prevention and treatment that included infections and treatment for children up to two years of age.

Katherine region is located in the tropical region of Australia (23.5^0^ South) that has a wet (October-March) and dry (April-September) season. Previous reports from tropical and subtropical countries indicate significant differences in antibiotic prescriptions and infectious presentations by season. [14–16] All three communities are accessible by road in the dry season but during the wet season two of the communities are often only accessible by air. [17] The three community populations ranged from ~300–1000 [18] for which there were ~215 births reported during the study period (personal communication from Data Custodian 2020). Health care is provided by RANs, Midwives and AHPs who are based at the community rPHC services and includes visiting Medical Officers and Allied Health Professionals.

Whilst acknowledging the diversity in Aboriginal and Torres Strait Islander peoples’ languages, culture and community populations, the provision and accessibility of PHC services and the prevalence of infections are not dissimilar across the Top End of Australia. [19]

### Participant recruitment and consent

From February to May 2016 locally trained Aboriginal Community Based Researchers went house to house to discuss the research project with families who potentially had children born between 1 January 2010 and 31 July 2014. Written informed consent was obtained from mothers/caregivers who agreed to having the electronic health records of their child’s PHC presentations in the first two years of life extracted for review. Participants were excluded if the child was born outside the study period, or if no electronic health data was recorded during their first two years of life.

Ethics approval was provided from the Human Research Ethics Committee (HREC) of the Northern Territory Department of Health and Menzies School of Health Research HREC (Reference number 2015–2525).

### Data extraction

From July to August 2016 an automated script was developed and modified by the local data custodian to extract data from the electronic health record into comma-separated value (CSV) files. Data variables used to merge the demographic CSV file with the reason for presentation and medication CSV files included a unique person identifier (hospital record number–HRN) and the date of the encounter. Other variables extracted included: Locality, Date of birth (DOB), Birth weight, Gestation, Clinical items (i.e. observations general, immunisation, review), Condition (i.e. diarrhoea, impetigo), Ear check left and right, Skin check, Medication type, Medication name, Medication indication, Medication instructions. One quarter of participants were randomly selected and their electronic health records manually reviewed to ensure all relevant data was adequately captured by the automated script.

### Data definitions

PHC encounters were presentations to the PHC service or a home visit by a health practitioner. Multiple encounters occurring on the same day were recorded as one encounter. Antibiotics were defined as prescribed if they were recorded in either the medication type, medication name or medication instructions fields. Infections were grouped into eight headings to allow for analysis of appropriateness of antibiotic prescription and defined by identifying string variables (listed below) in the Clinical items, Condition, Ear check left and right, Skin check or Medication indication fields.

Terms used in the electronic records to describe infections:

1. Diarrhoeal (diarrhoea, gastroenteritis, giardia, loose or runny stool)
2. Lower Respiratory Tract Infection (LRTI) (bronchitis, bronchiolitis, pneumonia, wheeze)
3. Upper Respiratory Tract Infection (URTI) (chest infection, cough, cold, congestion, croup, runny nose, sinusitis, sore throat, tonsillitis)
4. Acute otitis media (AOM) (otitis media, AOM)
5. Chronic suppurative otitis media (CSOM) (chronic otitis media, pus in ears)
6. Infected scabies (includes scabies diagnosis with either sores, impetigo or pustules)
7. Scabies (scabies diagnosis with no evidence of infection)
8. Skin Infection (abscess, boil, cellulitis, impetigo, pustules, sores, skin infection)

CARPA STM 7^th^ edition was used to determine whether antibiotic use was provided as indicated for the infectious classifications listed above (Table 1). [6]

**Table 1:** Antibiotics recommended by CARPA standard treatment manual [6] by infection.

	Diarrhoea	LRTI	URTI	AOM	CSOM	Infected Scabies	Skin infection
Amoxycillin	**-**	✓	✓	✓	✓	**-**	**-**
Amoxycillin- Clavulanate	**-**	✓	**-**	✓	**-**	**-**	**-**
Azithromycin	**-**	✓	**-**	**-**	**-**	**-**	**-**
Benzathine Penicillin	**-**	✓	✓	**-**	**-**	✓	✓
Cephalexin	**-**	✓	**-**	**-**	**-**	**-**	✓
Ciprofloxacin	**-**	**-**	**-**	✓	✓	**-**	**-**
Flucloxacillin	**-**	**-**	**-**	**-**	**-**	**-**	✓
Metronidazole	✓	**-**	**-**	**-**	**-**	**-**	**-**
Procaine Penicillin	**-**	✓	✓	**-**	**-**	**-**	✓
Sulfamethoxazole & Trimethoprim	✓	**-**	**-**	✓	**-**	✓	✓

Spectrum of activity was defined for Penicillins and Cephalosporins in accordance with the Therapeutic Guidelines (2017), [20] i) broad spectrum antibiotics included: Amoxycillin Clavulanate, Azithromycin, Ceftriaxone, Ciprofloxacin, Erythromycin, Metronidazole and Sulfamethoxazole & Trimethoprim, ii) moderate spectrum antibiotics included: Amoxycillin, Ampicillin and Cephalexin and iii) narrow spectrum antibiotics included: Benzathine Penicillin, Phenoxymethyl Penicillin, Flucloxacillin and Procaine Penicillin.

Low Birth Weight (LBW) was defined as <2.5kg, and term at birth was defined as i) Preterm <37 weeks completed gestation, ii) Term ≥37 weeks gestation [21].

### Data analysis

Outcome measures were: primary health care encounters, infectious presentations, antibiotic prescription rate, factors associated with antibiotic prescriptions and factors associated with appropriate antibiotic prescription. All data was analysed in STATA 16. [22]

Number of PHC encounters, infectious encounters and encounters recording an antibiotic prescription were reported as medians with interquartile ranges (IQRs) and stratified by age in years. Differences in median prescriptions by age, birth status and season were assessed by quantile (median) regression. Descriptive data for infectious encounters and infection type were reported as percentages. Antibiotic prescription rate for infectious encounters was reported as mean and standard deviation. Differences in the proportion of single infection or multiple infection encounters receiving antibiotics and receiving appropriate antibiotics was assessed via two tailed proportions test. Risk ratios (RR) were used to assess the risk of differing infections on antibiotic prescription–either alone as single infection encounters, alongside another as a multiple infection encounter, or in total.

For single infection encounters, a comparison of one particular infection was made with other single infections (i.e. single infection diarrhoea encounters vs. single infection non-diarrhoea encounters). Similarly, within multiple infection encounters all those containing a particular infection to those not containing this infection (i.e. multiple infection encounters containing diarrhoea vs. multiple infection encounters not containing diarrhoea). Finally, an overall risk comparing presence of a particular infection, regardless of single or multiple infection encounters, to infectious encounters without that particular infection (i.e. infectious encounters with diarrhoea vs. infectious encounters without diarrhoea). Logistic regression was used to assess the impact of short term (30-day period) infection recurrence on appropriateness of antibiotic prescriptions and reported as odds ratios (ORs).

## Results

Consent was obtained from 149 mothers to extract data from 196 child records. Seventy-two children were excluded from the analysis because; the records contained no data from the first two years of life (n = 5, 8%), the child was born after 31 July 2014 (n = 32, 44%), or the child was born prior to 1 January 2010 (n = 35, 49%). The 124 child records included represented ~69% of the eligible population, of which the majority (n = 81, 65%) were born at term weighing ≥2.5kg, 16 (13%) were term and LBW, 13 (10%) were preterm weighing ≥2.5kg and 14 (11%) were preterm and LBW (Table 2).

**Table 2:** Gestation and birthweight of children born 2010–2014.

	n = 124 (%)
Term & ≥2.5 kg	81 (65)
Term & LBW	16 (13)
Preterm & ≥2.5 kg	13 (10)
Preterm and LBW	14 (11)

**Fig 1:**
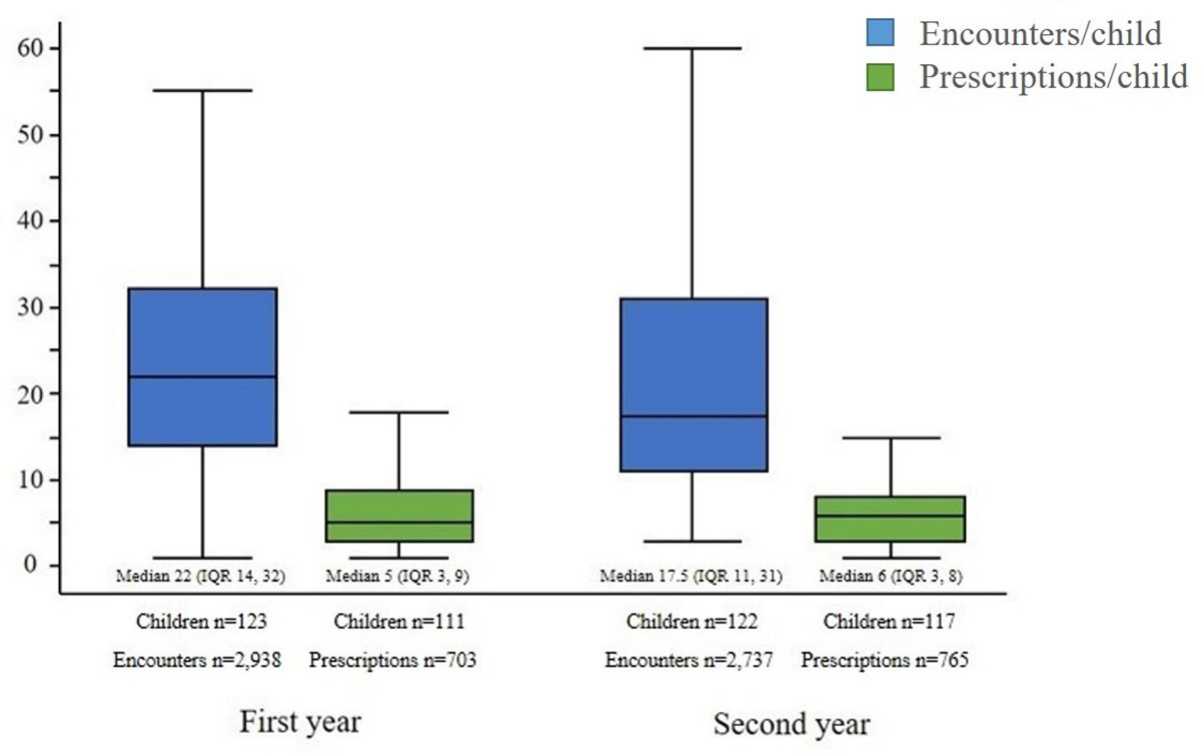
Median PHC encounters and antibiotic prescriptions per child, in the first and second year of life. (The figure is split to show first and second year of life. The blue boxes show the range of PHC encounters per child and the green boxes the range of recorded antibiotic prescriptions for the first and second year of life).

For the 124 children there were 5,675 PHC encounters in the first two years of life, median 41 (IQR 25.5, 64) encounters per child and 1,468 antibiotic prescriptions. In the first year of life, 123 (99%) children were seen with 2,938 PHC encounters (median 22, IQR 14, 32) for whom 111 (90%) had an infection recorded (median 6, IQR 3, 9) and 111 (90%) had antibiotics prescribed (median 5, IQR 3, 9) (Fig 1). In the second year of life, 122 (99%) children had 2,737 PHC encounters (median 17.5, IQR 11, 31) for whom 120 (99%) had an infection recorded (median 5.5 IQR 3, 9) and 117 (96%) had antibiotics prescribed (median 6, IQR 3, 8). No significant difference in PHC encounters, infectious encounters or antibiotic prescriptions was observed by age, birth status (LBW or preterm) or between seasons (wet or dry). The mean prescription rate for children was 5.99/person-year (95%CI 5.35–6.63).

**Fig 2:**
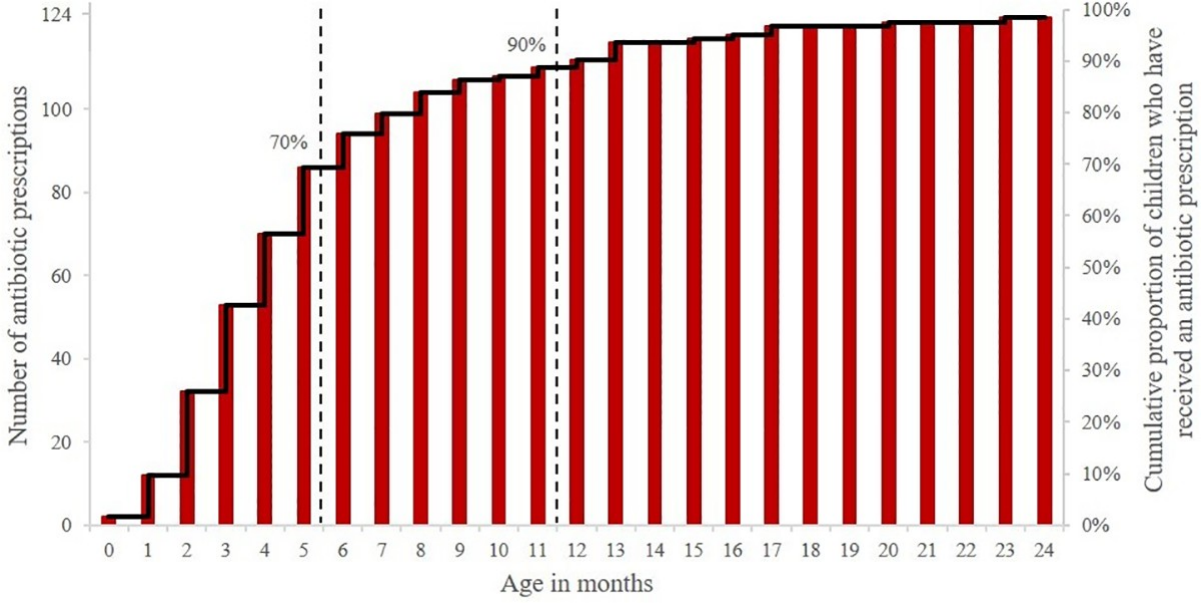
Prescriptions by spectrum of activity. (The total number of recorded antibiotic prescriptions by spectrum of activity, from left to right—Narrow, Moderate and Broad).

The majority of prescriptions were broad spectrum (n = 822, 56%) (Fig 2) of which Amoxycillin (n = 797, 54%) was the single most recorded prescription, followed by Benzathine Penicillin (n = 160, 11%).

**Fig 3:**
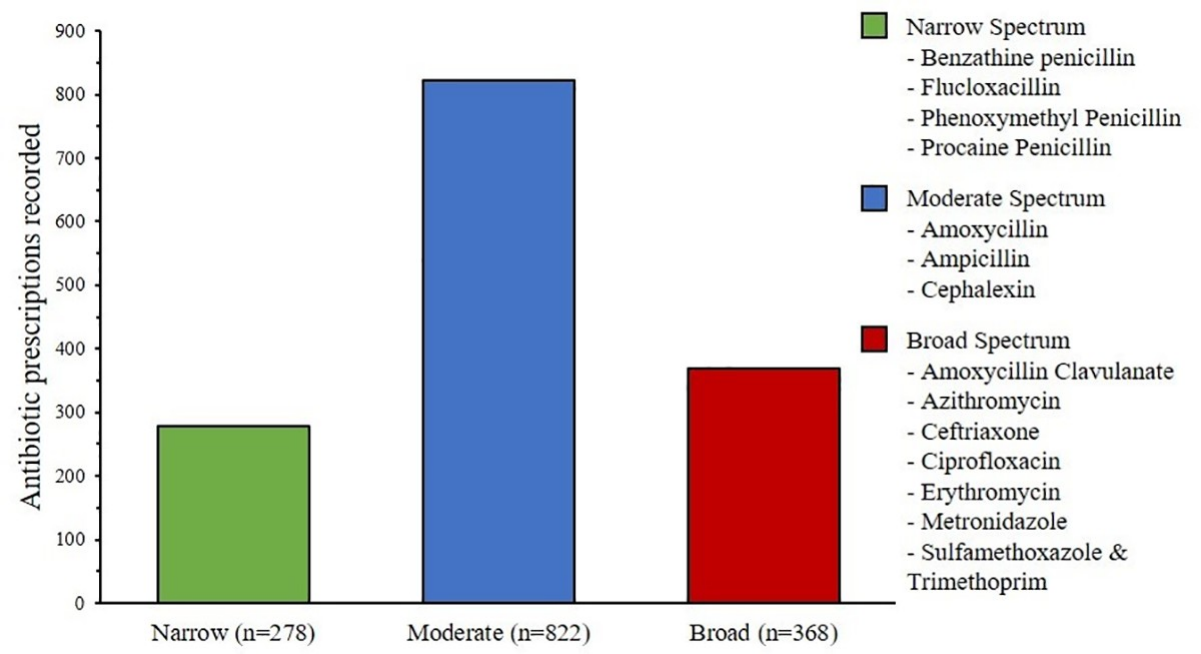
Cumulative incidence of first antibiotic prescription by age in months. (The bars represent the cumulative number of children who had an antibiotic prescription recorded by that age, while the line represents the cumulative proportion. Note two participants received an antibiotic in their first month of life, therefore the values do not start at zero. The dotted lines show the proportion of children who have received an antibiotic prescription prior to six and 12 months respectively).

For the 111 children who had an antibiotic prescription in the first year of life, the median age for the first prescription was four months (IQR 2–5). By 12 months of age, 90% of children had been prescribed an antibiotic (Fig 3).

Of the 5,675 PHC encounters, there were 1,542 (27%) with infections recorded, of which 1,342 (87%) encounters had a single infection and 200 (13%) encounters had multiple infections (two infections n = 181, three infections n = 18, four infections n = 1). For all infections (n = 1,777), the proportion of PHC encounters reporting single infections (n = 1,342 75%) was greater than those for multiple infections (Table 3). Within encounters recording multiple infections, AOM, CSOM and URTI were the most common co-infections.

**Table 3:** Number and percentage of single or multiple infections. (Column 2 shows the number and proportion of infections that occurred alone in a Single Infection Encounter. Column 3 shows the number and proportion of infections that occurred alongside at least one other infection in a Multiple Infection Encounter (note that as this column shows the number of infections (n = 435), the total value exceeds that of the number of multiple infection encounters (n = 200)). Column 4 shows the top three co-infections and the percentage of multiple infection encounters they were reported in for that infection).

	Single Infection n (%)	Multiple Infection n (%)	Top three co-infections (%)	Total n (%)
Diarrhoea	126 (78)	36 (22)	AOM (64), URTI (25) & CSOM (19)	**162 (9)**
LRTI	34 (87)	5 (13)	URTI (60), AOM (20) & CSOM (20)	**39 (2)**
URTI	210 (72)	82 (28)	AOM (68), CSOM (15) & Diarrhoea (11)	**292 (16)**
AOM	530 (78)	153 (22)	URTI (37), CSOM (25) & Skin (22)	**683 (38)**
CSOM	74 (54)	63 (46)	AOM (62), URTI (19) & Skin (14)	**137 (8)**
Infected Scabies*	14 (74)	5 (26)	AOM (80), Skin (80) & Diarrhoea (20)	**19 (1)**
Scabies	192 (88)	27 (12)	AOM (48), CSOM (30), URTI (19)	**219 (12)**
Skin infection	162 (72)	64 (28)	AOM (52), CSOM (11) & URTI (9)	**211 (12)**
**Total**	**1,342 (75)**	**435 (25)**	-	**1,777**

*Skin infection alongside infected scabies is for alternate skin infection presentations i.e. boils or cellulitis

Of the 1,542 encounters reporting an infection, 962 (62%) encounters had a record of an antibiotic prescription (mean prescription rate 0.69 ± 0.597). For the 1,342 single infection encounters, 795 (59%) recorded an antibiotic prescription, while of the 200 multiple infection encounters, 167 (84%) recorded an antibiotic prescription. Of the 795 recorded prescriptions for single infection encounters, 691 (87%) were appropriate and for the 167 multiple infection encounters, 161 (96%) were appropriate. The difference in proprtion of prescriptons and appropriateness of prescriptions between single and multiple infection encounters was statistically significant (p<0.0001 & p = 0.0002 respectively).

Among encounters with a single infection recorded, AOM had the highest proportion of antibiotic prescriptions (83%) and accounted for more than half of the total antibiotic prescriptions (n = 439, 56%) (Table 4). Furthermore, all but eight (2%) AOM prescriptions were considered appropriate. Of the scabies infections which recorded antibiotic prescriptions 31 (63%) received Benzathine Penicillin. Other antibiotic prescriptions recorded for scabies included: Amoxycillin; n = 7, Amoxycillin/Clavulanate, Sulfamethoxazole & Trimethoprim and Flucloxacillin; n = 3, Azithromycin, Cephalexin and Procaine Penicillin; n = 1.

**Table 4:** Antibiotic appropriateness by infection (single infection encounters only).

	Antibiotic Prescribed n (%)	Aligned Prescription n (%)	Non Aligned Prescription n (%)
Diarrhoea (n = 126)	30 (24)	24/30 (80)	6/30 (20)
LRTI (n = 34)	24 (71)	22/24 (92)	2/24 (8)
URTI (n = 210)	128 (61)	104/128 (81)	24/128 (19)
AOM (n = 530)	439 (83)	431/439 (98)	8/439(2)
CSOM (n = 74)	42 (57)	37/42 (88)	5/42 (12)
Infected scabies (n = 19)	12 (86)	10/12 (83)	2/12 (17)
Scabies (n = 192)	49 (26)	0/49 (0)	49/49 (100)
Skin Infection (n = 162)	71 (44)	63/71 (89)	8/71 (11)
**Total (n = 1,342)**	**795 (59)**	**691/795 (87)**	**70/795 (13)**

PHC encounters with AOM recorded had a significantly greater risk of antibiotic prescription than for any other infection, regardless if presenting as a single infection (RR 3.32, 95% CI 2.72, 4.05) or as part of a multiple infection encounter (1.31 95% CI 0.99, 1.75) (Table 5). Antibiotics were significantly less likely to be prescribed if the encounter recorded either diarrhoea or a skin infection.

**Table 5:** Number and percentage of single and multiple infections and the risk ratio for having an antibiotic prescribed compared to other infections.

	Single infections for which an antibiotic was prescribed n = 795 (%) RR (95% CI)		Multiple infections for which an antibitoic was prescribed n = 167 (%) RR (95% CI)		Any infection for which an antibiotic was prescribed n = 962 (%) RR (95% CI)	
Diarrhoea	30 (4)	0.22 (0.15, 0.32)	24 (14)	0.40 (0.22, 0.71)	**54 (6)**	**0.30 (0.22, 0.41)**
LRTI	24 (3)	1.65 (0.80, 3.43)	3 (2)	0.30 (0.05, 1.71)	**27 (3)**	**1.36 (0.69, 2.66)**
URTI	128 (16)	1.07 (0.83, 1.39)	73 (44)	1.60 (0.90, 2.87)	**201 (21)**	**1.33 (1.06, 1.67)**
AOM	439 (55)	3.32 (2.72, 4.05)	133 (80)	1.31 (0.99, 1.75)	**572 (59)**	**3.11 (2.61, 3.7)**
CSOM	42 (5)	0.90 (0.58, 1.41)	54 (32)	1.19 (0.65, 2.16)	**96 (10)**	**1.41 (0.99, 2.01)**
Infected Scabies	12 (2)	4.13 (0.93, 18.37)	4 (3)	0.79 (0.09, 6.85)	**16 (2)**	**3.22 (0.94, 10.99)**
Scabies	49 (6)	0.24 (0.17, 0.32)	23 (14)	1.14 (0.42, 3.07)	**72 (8)**	**0.30 (0.23, 0.38)**
Skin Infection	71 (9)	0.54 (0.40, 0.72)	35 (21)	0.49 (0.30, 0.81)	**106 (11)**	**0.61 (0.47, 0.78)**

Almost two thirds (n = 954, 65%) of antibiotic prescriptions were prescribed for infections that aligned with what was recommended by CARPA standard treatment manual (Table 6). Of the encounters where a prescription without an infection was recorded (n = 398, 27%), Amoxycillin was the most common prescription (n = 176, 44%). Amoxycillin was also the most frequent antibiotic presribed for infections that aligned with CARPA (76%).

**Table 6:** Prescriptions administered when indicated in accordance with CARPA.

	Aligned with CARPA n = 954 (%)	Not aligned n = 116 (%)	No infection recorded n = 398 (%)	Total Prescriptions n = 1468 (%)
Amoxycillin	607 (76)	14 (2)	176 (22)	**797 (54)**
Amoxycillin Clavulanate	69 (54)	34 (26)	26 (20)	**129 (9)**
Ampicillin	0 (0)	0 (0)	1 (100)	**1 (0)**
Azithromycin	0 (0)	3 (75)	1 (25)	**4 (0)**
Benzathine Penicillin	76 (48)	34 (21)	50 (31)	**160 (11)**
Ceftriaxone	0 (0)	5 (45)	6 (55)	**11 (1)**
Cephalexin	7 (29)	10 (42)	7 (29)	**24 (2)**
Ciprofloxacin	107 (74)	1 (1)	37 (26)	**145 (10)**
Erythromycin	0 (0)	1 (33)	2 (67)	**3 (0)**
Flucloxacillin	18 (49)	5 (14)	14 (38)	**37 (3)**
Metronidazole	30 (58)	2 (4)	20 (39)	**52 (4)**
Phenoxymethyl Penicillin	0 (0)	2 (100)	0 (0)	**2 (0)**
Procaine Penicillin	29 (37)	2 (3)	48 (61)	**79 (5)**
Sulfamethoxazole & Trimethoprim	11 (46)	3 (13)	10 (42)	**24 (2)**

**Fig 4:**
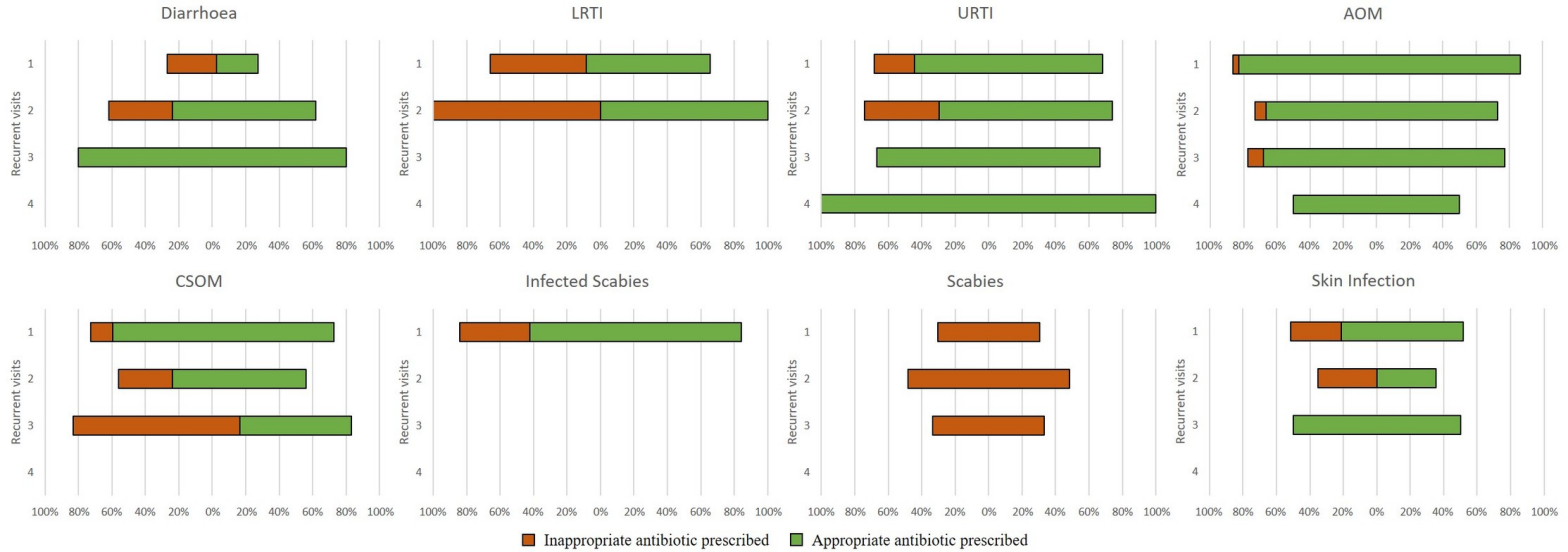
The proportion of infectious encounters which received an antibiotic prescription by infection recurrence within a one calender month time period. (The top bar for each graph shows the proportion of participants who presented with the respective infection for the first time in a calendar month and recorded an antibiotic prescription which was either appropriate (green) or inappropriate (orange). The second bar down shows the proportion of children who presented for that infection a second time in a calendar month and recorded an antibiotic prescription. The third and fourth bars show the same for the third and fourth presentations within a calendar month respectively. Absent bars may represent either there was no recurrent visit, or recurrent visits did not receive any antibiotic).

Looking to participants who presented with multiple episodes of the same infection in a one calender month time span, the prescription pattern and appropriateness of prescriptions differed between infections. The majority of first time AOM infectious presentations (n = 536) received an appropriate antibiotic prescription (n = 454, 85%) (Table 7). The proportion of appropriate prescriptions declined significantly with recurrent visits (OR 0.54, 95% CI 0.39, 0.75, p<0.001, R^2^ = 0.0196) (Fig 4). Diarrhoeal infections displayed the opposite trend, with the first visit having few presciptions (27%) and low appropriateness (13%) which increased significantly with recurrence (OR 5.27, 95% CI 2.39, 11.6, p<0.001, R^2^ = 0.1229).

**Table 7:** Number of repeated infectious encounters over calender month, the proportion which received antibiotics and the appropriateness of antibiotics.

		Recurrent encounters within calender month				
		1	2	3	4	OR (95% CI)
Diarrhoea	Presentation	136	21	5		5.27 (2.39, 11.6)
	Treated	37 (27)	13 (62)	4 (80)		
	*Appropriate*	*17 (13)*	*9 (43)*	*4 (80)*		
LRTI	Presentation	35	4			1.69 (0.21, 13.5)
	Treated	23 (66)	4 (100)			
	*Appropriate*	*13 (37)*	*2 (50)*			
URTI	Presentation	261	27	3	1	1.08 (0.59, 1.97)
	Treated	178 (68)	20 (74)	2 (67)	1 (100)	
	*Appropriate*	*147 (56)*	*14 (52)*	*2 (67)*	*1 (100)*	
AOM	Presentation	536	123	22	2	0.54 (0.39, 0.75)
	Treated	464 (87)	90 (73)	17 (77)	1 (50)	
	*Appropriate*	*454 (85)*	*86 (70)*	*16 (73)*	*1 (50)*	
CSOM	Presentation	106	25	6		0.42 (0.21, 0.82)
	Treated	77 (73)	14 (56)	5 (83)		
	*Appropriate*	*70 (66)*	*10 (40)*	*2 (33)*		
Infected Scabies	Presentation	19				-
	Treated	16 (84)				
	*Appropriate*	*12 (63)*				
Scabies	Presentation	187	29	3		-
	Treated	57 (30)	14 (48)	1 (33)		
	*Appropriate*	*0*	*0*	*0*		
Skin infection	Presentation	192	17	2		0.6 (0.23, 1.6)
	Treated	99 (52)	6 (35)	1 (50)		
	*Appropriate*	*70 (37)*	*3 (18)*	*1 (50)*		

## Discussion

Australian Aboriginal children in remote communities had exceptionally high levels of antibiotic prescriptions in comparison to Australian values reported previously. [2, 5, 8, 23] By 12 months of age, 90% of Aboriginal children in this study had received at least one antibiotic prescription, comparable to that previously reported in East Arnhem (95%) [9] and six of eight low- and middle-income countries (LMICs) in the MAL-ED study (80–100%), [24] but significantly higher than reported in a regional centre in Victoria (50%) [23] and previous reports from Europe (18–55%). [25] The prescription rate among Aboriginal children per person year (5.99, 95% CI 5.35, 6.63) in this study was six times higher than that reported for children in regional Victoria (0.92, 95% CI 0.83, 1.02) [23] and slightly higher than the average 4.9 antibiotic courses per person year in the MAL-ED countries. [24]

Similarities in antibiotic prescriptions can be seen between the current setting and LMICs. A recent report using retrospective data from eight LMICs estimated 62.3% and 63.2% of children aged zero and one years respectively presented to a health care service with infection and received an antibiotic prescription—similar to this study in which 62% of infectious encounters recorded an antibiotic prescription. [26] Comparing specific infections and antibiotic prescriptions, in the current study 33% of diarrhoeal episodes recorded antibiotic prescriptions, similar to the average reported across countries in the MAL-ED study. [24] Respiratory infections however were significantly more likely to receive antibiotics in the current study than any site in the MAL-ED study, with 67% receiving antibiotics compared to 62.8% for the highest site (Haydom, Tanzania) and 43.5% for the MAL-ED sites overall. [24] In the study by Fink et al. (2019) mean antibiotic prescription per sick child visit was 0.66 (SD 0.547) and 0.67 (SD 0.553) in the first and second year of life, comparable to the overall 0.69 (SD 0.597) in the current study. [26]

Amoxycillin was the most commonly prescribed antibiotic, accounting for more than half the total prescriptions (54%). This is higher than usage reported through the PBS (43.7%) [8] and through parental or community reporting (55%—this figure includes Ampicillin and Amoxycillin/Clavulanate). [23] It is noteworthy that in this remote setting, the use of narrow spectrum antibiotics was significantly higher than that reported previously through the PBS (19% vs 9%). [27] High prescription rates of moderate and broad spectrum antibiotics contribute more to the spread of antibiotic resistance due to cross resistance than do narrow spectrum antibiotics. [27, 28] Thus, although the prescription rate is higher in this remote setting, the ratio of broad to narrow spectrum antibiotic prescriptions is lower than reported in urban settings.

The high rates of antibiotic prescription are attributable to an underlying burden of disease that is substantially higher in Aboriginal children than non-Aboriginal children. [29–31] Children presented with an infection a median 6 (IQR 3–9) and 5.5 (IQR 3–9) times in their first and second year of life respectively, which is consistent with the mean antibiotic prescription rate noted (5.99, 95% CI 5.35, 6.63). If this prescription rate continued it would be similar to LMICs which reported a mean 36 (95%CI 33.8, 38.3) sick visits by the age of five years. [26] In the current study, AOM was the most common infection, recorded alone in 30% of infectious encounters, and alongside other infections in an additional 8%. The majority of encounters with AOM alone (n = 530) had an antibiotic prescription recorded (n = 439, 83%), which accounted for more than half the total prescriptions recorded among single infection encounters (n = 783, 56%). Of the encounters which recorded an Amoxycillin prescription 60% (n = 478) also recorded AOM. In comparison to the wider Australian community, 15% of Amoxycillin prescriptions nationally were used to treat AOM. [2]

Though single infection encounters were more common (86% of infectious encounters), multiple infection encounters accounted for 25% of the total infection burden. Multiple infection encounters were significantly more likely to receive antibiotics compared to single infection encounters (84% vs 59%) and for these to be appropriate antibiotics (98% vs 91%). AOM and URTIs were the most common concurrent infections, present in 34% and 18% of multiple infection encounters respectively. However, the presence of any one particular infection did not increase the likelihood of receiving antibiotics which is likely due to the high prescription rate already present. These aspects together highlight the role the underlying burden of disease plays in antibiotic prescription rates in remote communities. Cunningham et al. (2019) estimated a 6% absolute reduction in antibiotic prescriptions if skin infections were eliminated in their studied communities. [9] Our data indicate an absolute reduction of 4% in antibitoic prescription rates if skin infections were eliminated. If AOM were eliminated in these three communities however, we estimate a 30% absolute reduction.

This study is the first to report on appropriateness of prescriptions within remote Aboriginal Australian communities, and previous reports have noted the need for such data. [2, 9, 19] In this study, 65% of prescriptions had recorded infections that aligned with CARPA guidelines. Only 8% of prescriptions had an infection recorded which did not align, and 27% of prescriptions had no infection recorded, significantly lower than that reported in AURA 2017. (2) In the case of non-infected scabies, for which one quarter of single infection encounters received an antibiotic, 63% of these encounters received Benzathine Penicillin which is indicated in cases of infected scabies. We are unable to differentiate if this was inappropriate use of Benzathine Penicillin or if scabies that were infected were not recorded correctly in the electronic health records.

Appropriateness of prescriptions aside, the high rate of prescriptions is a potential issue in and of itself. Antibiotic use in infancy is associated with a long-term shift in the gut microbiome which may further increase the risk of non-communicable chronic disease (NCDs) in this vulnerable population. [32] According to a 2016 report, the burden of disease among Aboriginal Australians is 2.3 times that of non-Aboriginal Australians, with 70% of this accounted for by chronic disease. [33] Around one-third of this is considered preventable by modifying classic risk factors (ie. Tobacco, body mass, diet, physical inactivity) [33] however, the current study suggests an additional avenue for NCD mitigation, by addressing the underlying burden of infectious disease in infancy and subsequent antibiotic prescriptions which alter the gut microbiome.

The temporal dimension of antibiotic prescriptions is often unexplored. This study provides a comprehensive description of infections and the appropriateness of antibiotic prescriptions. It is noteworthy that for less potentially serious infections, (i.e. diarrhoea, URTIs) antibiotic prescription at the first visit tended to be low but increasing over time with recurrent encounters. This may indicate that infections were thought to be parasitic or viral and required other first line treatment measures.

Legislative requirements placed on nurses and Aboriginal Health Practitioners to adhere to the guidelines when supplying and administering antibiotics is an effective mechanism to limit inappropriate antibiotic use in this setting. [34] For the more prominent infections (AOM and CSOM) appropriate prescriptions were high initially but dwindled over time with recurrence. However, those that did receive an antibiotic prescription were more likely to be receiving a repeat prescription. This is likely a reflection of the context these communities are in. Both AOM and CSOM are endemic within such communities and have significant sequalae. Thus, health practitioners have greater motivation to treat promptly and offer longer courses of antibiotics to ensure successful treatment.

A major strength of the current study is the level of clinical information available within the electronic health record. The majority (73%) of antibiotic prescriptions had an infection listed, allowing for more accurate determination of appropriateness in comparison to AURA 2017 (23.5% had infections recorded). [2] The automated script used to withdraw relevant data from the electronic health record is a significant advantage over manual review of all progress notes. Once all data fields were entered into the automated script, less than 1% of observations were found to have additional data within the progress notes. This gives a strong base from which to develop ongoing surveillance processes to monitor antimicrobial stewardship. One major limitation of this study is the binary view taken to determine appropriateness of antibiotic prescriptions. This assumes that all clinically relevant data has been entered by the frontline staff in extractable sections (not progress notes), and that any infections present were at a level which required antibiotic intervention. Another potential limitation is missing data, as children may have travelled to other communities, collected a prescription there and on returning home presented for repeats at their home PHC–thus contributing to the number of ‘inappropriate’ prescriptions.

## Conclusion

Antibiotic prescription rates among Australian Aboriginal children living in remote communities are significantly higher than that reported for non-Aboriginal Australians. The majority of antibiotic prescriptions are appropriate and driven by a higher underlying burden of disease thus, the consequences of a potential rise in antibiotic resistance are significant. Compared to urban centres, the ratio of broad to narrow spectrum antibiotic prescriptions is positive, suggesting awareness of appropriate antimicrobial stewardship in these communities. However, the high prescription rates in infancy may have some contribution to chronic disease later in life. Within these communities a focus on promoting the underlying determinants of health is critical to reduce the burden of disease which underpins antibiotic prescription rates.

## Supporting information

S1 Appendix Number of prescriptions recorded for each antibiotic and indications present. Green boxes represent ‘appropriate’.
(DOCX)

S1 Data (JPG)

## References

[1] Aminov RI. A brief history of the antibiotic era: lessons learned and challenges for the future. Frontiers in microbiology. 2010;1. 10.3389/fmicb.2010.00134 PMC3109405 21687759

[2] Australian Commission on Safety and Quality in Health Care. AURA 2017: second Australian report on antimicrobial use and resistance in human health. Sydney: ACSQHC; 2017.

[3] O’Neill J. Antimicrobial resistance: tackling a crisis for the health and wealth of nations. Review on antimicrobial resistance. 2014:1–16.

[4] Cecchini M, Langer J, Slawomirski L. Antimicrobial Resistance in G7 Countries and Beyond: Economic Issues, Policies and Options for Action. Paris: Organization for Economic Co-operation and Development 2015.

[5] Australian Commission on Safety and Quality in Health Care. AURA 2016: first Australian report on antimicrobial use and resistance in human health. Sydney: ACSQHC; 2016.

[6] Central Australian Rural Practitioners Association. Central Australian Rural Practitioners Association standard treatment manual. 7 ed Alice Springs, NT: Centre for Remote Health: CARPA; 2017.

[7] Australian Government Department of Health. S100 Remote Area Aboriginal Health Services (RAAHS) Program Information Sheet: Department of Health; 2019 [updated 9/4/2019. Available from: https://www1.health.gov.au/internet/main/publishing.nsf/Content/health-pbs-indigenous-info.

[8] Gadzhanova S, Roughead E. Prescribed antibiotic use in Australian children aged 0–12 years. Australian family physician. 2016;45(3):134 27052051

[9] Cuningham W, McVernon J, Lydeamore MJ, Andrews RM, Carapetis J, Kearns T, et al High burden of infectious disease and antibiotic use in early life in Australian Aboriginal communities. Australian and New Zealand journal of public health. 2019;43(2):149–55. 10.1111/1753-6405.12876 30727032

[10] Kearns T, Clucas D, Connors C, Currie BJ, Carapetis JR, Andrews RM. Clinic attendances during the first 12 months of life for Aboriginal children in five remote communities of northern Australia. PLoS One. 2013;8(3):e58231 10.1371/journal.pone.0058231 23469270 PMC3585931

[11] McMeniman E, Holden L, Kearns T, Clucas DB, Carapetis JR, Currie BJ, et al Skin disease in the first two years of life in Aboriginal children in East Arnhem Land. Australasian Journal of Dermatology. 2011;52(4):270–3. 10.1111/j.1440-0960.2011.00806.x 22070701

[12] Morris PS, Leach AJ, Silberberg P, Mellon G, Wilson C, Hamilton E, et al Otitis media in young Aboriginal children from remote communities in Northern and Central Australia: a cross-sectional survey. BMC pediatrics. 2005;5(1):27. 16033643 10.1186/1471-2431-5-27 PMC1187897

[13] Murray R. Prescribing issues for Aboriginal people. Australian Prescribing. 2003;26:106–9.

[14] Farooqui HH, Selvaraj S, Mehta A, Heymann DL. Community level antibiotic utilization in India and its comparison vis-à-vis European countries: Evidence from pharmaceutical sales data. PloS one. 2018;13(10):e0204805 10.1371/journal.pone.0204805 30332450 PMC6192587

[15] Greer RC, Intralawan D, Mukaka M, Wannapinij P, Day NP, Nedsuwan S, et al Retrospective review of the management of acute infections and the indications for antibiotic prescription in primary care in northern Thailand. BMJ open. 2018;8(7):e022250 10.1136/bmjopen-2018-022250 30061442 PMC6067334

[16] Shah M, Kathiiko C, Wada A, Odoyo E, Bundi M, Miringu G, et al Prevalence, seasonal variation, and antibiotic resistance pattern of enteric bacterial pathogens among hospitalized diarrheic children in suburban regions of central Kenya. Tropical medicine and health. 2016;44(1):39. 27942243 10.1186/s41182-016-0038-1 PMC5126808

[17] Sunrise Health. Sunrise Sites 2016 [Available from: http://www.sunrise.org.au/sunrise/sunrisesites.htm.

[18] Australian Bureau of Statistics. Census 2011. Canberra: ABS; 2011.

[19] Bowen AC, Daveson K, Anderson L, Tong SY. An urgent need for antimicrobial stewardship in Indigenous rural and remote primary health care. The Medical Journal of Australia. 2019;211(1):9–11. e1. 10.5694/mja2.50216 31155725

[20] Therapeutic Guidelines Limited. eTG Complete Nth Melbourne, Vic.: Therapeutic Guidelines Ltd.; 2005 [

[21] United Nations Children’s Fund, World Health Organization. Low Birthweight: Country, regional and global estimates. New York: UNICEF; 2004.

[22] StataCorp. Stata statistical software: Release 16. College Station, TX: StataCorp LLC; 2019.

[23] Anderson H, Vuillermin P, Jachno K, Allen KJ, Tang ML, Collier F, et al Prevalence and determinants of antibiotic exposure in infants: A population‐derived Australian birth cohort study. Journal of Paediatrics and Child Health. 2017. 10.1111/jpc.13616 28749577

[24] Rogawski ET, Platts-Mills JA, Seidman JC, John S, Mahfuz M, Ulak M, et al Use of antibiotics in children younger than two years in eight countries: a prospective cohort study. Bulletin of the World Health Organization. 2017;95(1):49 10.2471/BLT.16.176123 28053364 PMC5180352

[25] Stam J, van Stuijvenberg M, Grüber C, Mosca F, Arslanoglu S, Chirico G, et al Antibiotic use in infants in the first year of life in five European countries. Acta paediatrica. 2012;101(9):929–34. 10.1111/j.1651-2227.2012.02728.x 22691104

[26] Fink G, D'Acremont V, Leslie HH, Cohen J. Antibiotic exposure among children younger than 5 years in low-income and middle-income countries: a cross-sectional study of nationally representative facility-based and household-based surveys. The Lancet Infectious Diseases. 2019. 10.1016/S1473-3099(19)30572-9 31843383

[27] Drug Utilisation Sub Committee (DUSC). Antibiotics: PBS/RPBS utilisation. Canberra; 2014.

[28] Collignon PJ. 11: Antibiotic resistance. Medical journal of Australia. 2002;177(6):325–31. 12225283 10.5694/j.1326-5377.2002.tb04794.x

[29] Read AW, Gibbins J, Stanley FJ, Morich P. Hospital admissions before the age of 2 years in Western Australia. Archives of disease in childhood. 1994;70(3):205–10. 10.1136/adc.70.3.205 8135564 PMC1029743

[30] McAuley K, McAullay D, Strobel NA, Marriott R, Atkinson DN, Marley JV, et al Hospital utilisation in indigenous and non-indigenous infants under 12 months of age in Western Australia, prospective population based data linkage study. PloS one. 2016;11(4):e0154171 10.1371/journal.pone.0154171 27120331 PMC4847930

[31] Australian Institute of Health and Welfare. National Partnership Agreement on Indigenous Early Childhood Development: Second Annual Report on Health Performance Indicators. AIHW Canberra; 2015.

[32] Korpela K, Salonen A, Virta LJ, Kekkonen RA, Forslund K, Bork P, et al Intestinal microbiome is related to lifetime antibiotic use in Finnish pre-school children. Nature communications. 2016;7:10410 10.1038/ncomms10410 26811868 PMC4737757

[33] Al-Yaman F. The Australian Burden of Disease Study: impact and causes of illness and death in Aboriginal and Torres Strait Islander people, 2011. Public Health Res Pract. 2017;27(4):e2741732. 10.17061/phrp2741732 29114712

[34] Northern Territory Government Department of Health. Environmental health—Public Health: Medicines and poisons notices: Department of Health,; 2019 [updated 7/12/2019. Available from: https://health.nt.gov.au/professionals/environmental-health/medicines-and-poisons-notices.

