# Correction: Antibiotic use for Australian Aboriginal children in three remote Northern Territory communities

**DOI:** 10.1371/journal.pone.0233533

**Published:** 2020-05-14

**Authors:** Timothy Howarth, Raelene Brunette, Tanya Davies, Ross M. Andrews, Bhavini K. Patel, Steven Tong, Federica Barzi, Therese M. Kearns

There is an error in affiliation 1 for authors Timothy Howarth and Therese M. Kearns, in affiliation 4 for author Ross M. Andrews, and in affiliation 7 for author Federica Barzi. The correct affiliation 1 is: Child Health, Menzies School of Health Research, Charles Darwin University, Darwin, Northern Territory, Australia. The correct affiliation 4 is: Tropical Health, Menzies School of Health Research, Charles Darwin University, Brisbane, Queensland, Australia. The correct affiliation 7 is: Wellbeing and Preventable Chronic Diseases, Menzies School of Health Research, Charles Darwin University, Darwin, Northern Territory, Australia.
