# Supplementary material for: Antibiotic use for Australian Aboriginal children in three remote Northern Territory communities
Source: PLoS One. 2020 Apr 17;15(4):e0231798. doi: 10.1371/journal.pone.0231798 (PMC7164616; doi:10.1371/journal.pone.0231798)
Supplement: S1 Appendix — Green boxes represent ‘appropriate’. (DOCX) [file pone.0231798.s001.docx]

**Appendix 1. Number of prescriptions recorded for each antibiotic and indications present.** Green boxes represent ‘appropriate’

|  | Multiple Indication | Diarrhoea | LRTI | URTI | AOM | CSOM | Infected scabies | Non-infected Scabies | Skin Infection | No Infection | **Total (%)** |
| --- | --- | --- | --- | --- | --- | --- | --- | --- | --- | --- | --- |
| Amoxycillin | 113 (53) | 4 (13) | 6 (22) | 88 (65) | 383 (81) | 16 (30) | 1 (8) | 7 (14) | 3 (4) | 176 (44) | **797 (14)** |
| Amoxycillin Clavulanate | 26 (12) | 1 (3) | 2 (7) | 23 (17) | 37 (8) | 5 (9) | 0 (0) | 3 (6) | 6 (8) | 26 (7) | **129 (2)** |
| Ampicillin | 0 (0) | 0 (0) | 0 (0) | 0 (0) | 0 (0) | 0 (0) | 0 (0) | 0 (0) | 0 (0) | 1 (0) | **1 (0)** |
| Azithromycin | 0 (0) | 0 (0) | 0 (0) | 2 (1) | 0 (0) | 0 (0) | 0 (0) | 1 (2) | 0 (0) | 1 (0) | **4 (0)** |
| Benzathine Penicillin | 15 (7) | 0 (0) | 4 (15) | 5 (4) | 1 (0) | 1 (2) | 10 (77) | 31 (62) | 43 (57) | 50 (13) | **160 (3)** |
| Ceftriaxone | 0 () | 0 (0) | 4 (15) | 0 (0) | 1 (0) | 0 (0) | 0 (0) | 0 (0) | 0 (0) | 6 (2) | **11 (0)** |
| Cephalexin | 2 (1) | 1 (3) | 1 (4) | 1 (1) | 6 (1) | 2 (4) | 0 (0) | 1 (2) | 3 (4) | 7 (2) | **24 (0)** |
| Ciprofloxacin | 39 (18) | 0 (0) | 1 (4) | 1 (1) | 38 (8) | 29 (54) | 0 (0) | 0 (0) | 0 (0) | 37 (9) | **145 (3)** |
| Erythromycin | 0 (0) | 1 (3) | 0 (0) | 0 (0) | 0 (0) | 0 (0) | 0 (0) | 0 (0) | 0 (0) | 2 (1) | **3 (0)** |
| Flucloxacillin | 2 (1) | 0 (0) | 0 (0) | 0 (0) | 0 (0) | 0 (0) | 2 (15) | 3 (6) | 16 (21) | 14 (4) | **37 (1)** |
| Metronidazole | 4 (2) | 24 (77) | 0 (0) | 1 (1) | 2 (0) | 0 (0) | 0 (0) | 0 (0) | 1 (1) | 20 (5) | **52 (1)** |
| Phenoxymethyl Penicillin | 1 (0) | 0 (0) | 0 (0) | 0 (0) | 1 (0) | 0 (0) | 0 (0) | 0 (0) | 0 (0) | 0 (0) | **2 (0)** |
| Procaine Penicillin | 4 (2) | 0 (0) | 9 (33) | 15 (11) | 0 (0) | 1 (2) | 0 (0) | 1 (2) | 1 (1) | 48 (12) | **79 (1)** |
| Sulfamethoxazole & Trimethoprim | 8 (4) | 0 (0) | 0 (0) | 0 (0) | 1 (0) | 0 (0) | 0 (0) | 3 (6) | 2 (3) | 10 (3) | **24 (0)** |
| **Total** | **214 (15)** | **31 (2)** | **27 (2)** | **136 (9)** | **470 (32)** | **54 (4)** | **13 (1)** | **50 (3)** | **75 (5)** | **398 (27)** | **1468 (100)** |
