# Supplementary figures and images for: Antibiotic use for Australian Aboriginal children in three remote Northern Territory communities
Source: PLoS One. 2020 Apr 17;15(4):e0231798. doi: 10.1371/journal.pone.0231798 (PMC7164616; doi:10.1371/journal.pone.0231798)

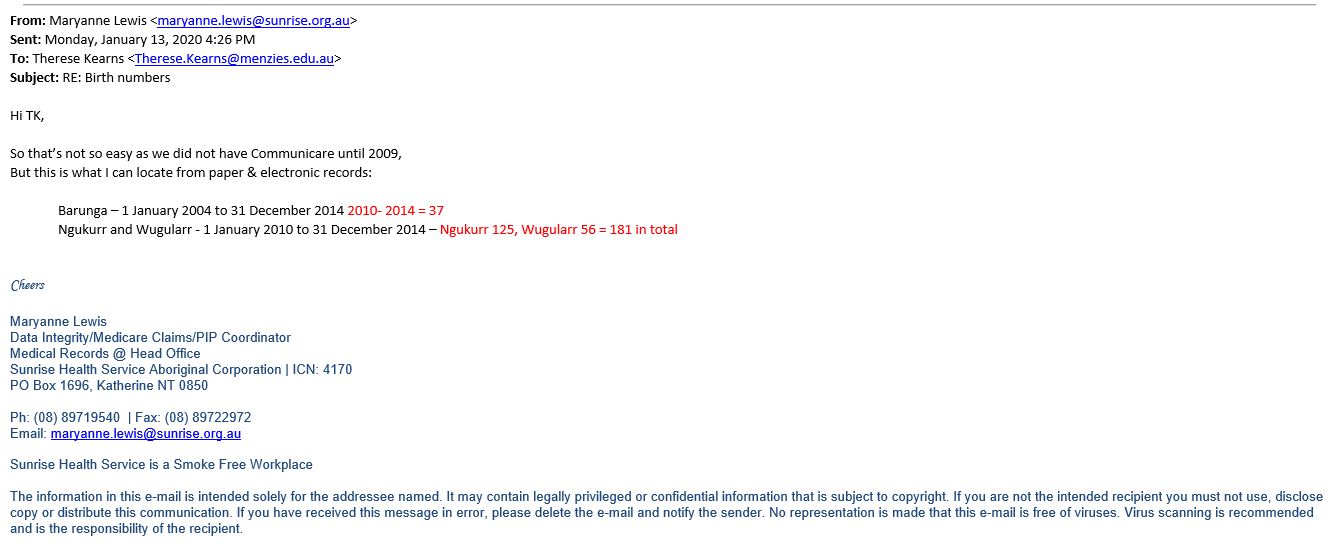

Supplement: S1 Data — (JPG) [file pone.0231798.s002.JPG]
